# The early impact of COVID-19 on primary care psychological therapy services: A descriptive time series of electronic healthcare records

**DOI:** 10.1016/j.eclinm.2021.100939

**Published:** 2021-06-04

**Authors:** Clarissa Bauer-Staeb, Alice Davis, Theresa Smith, Wendy Wilsher, David Betts, Chris Eldridge, Emma Griffith, Julian Faraway, Katherine S. Button

**Affiliations:** a Department of Psychology, University of Bath, , Claverton Down, Bath BA2 7AY, United Kingdom; b Department of Mathematical Sciences, University of Bath, Bath, United Kingdom; c Mayden, Bath, United Kingdom; d Avon and Wiltshire Mental Health Partnership NHS Trust, Bath, United Kingdom

## Abstract

**Background:**

There are growing concerns about the impact of the COVID-19 pandemic on mental health. With government-imposed restrictions as well as a general burden on healthcare systems, the pandemic has the potential to disrupt the access to, and delivery of, mental healthcare.

**Methods:**

Electronic healthcare records from primary care psychological therapy services (Improving Access to Psychological Therapy) in England were used to examine changes in access to mental health services and service delivery during early stages of the COVID-19 pandemic. A descriptive time series was conducted using data from five NHS trusts to examine patterns in referrals to services (1st January 2019 to 24th May 2020) and appointments (1st January 2020 to 24th May 2020) taking place.

**Findings:**

The number of patients accessing mental health services dropped by an average of 55% in the early weeks after the March 2020 lockdown was announced, reaching a maximum reduction of 74% in the initial 3 weeks after lockdown in the UK, which gradually recovered to a 28% reduction by May. We found some evidence suggesting changes in the sociodemographic and clinical characteristics of referrals. Despite a reduction in access, the impact on appointments appeared limited with service providers shifting to remote delivery of care.

**Interpretation:**

Services appeared to adapt to provide continuity of care in mental healthcare. However, patients accessing services reduced, potentially placing a future burden on service. Despite the observational nature of the data, the present study can inform the planning of service provision and policy.

**Funding:**

AD and TS were funded by Innovate UK (KTP #11,105).

**Research in context**

**Evidence before this study**

Google Scholar was searched using the terms “COVID-19″ and “mental health services”. Due to the novelty of the research few peer-reviewed articles had been published (pre-prints excluded) at the time of data analysis. Since then, research surveying mental health staff reports rapid innovation in services to adapt to COVID-19, with an emphasis on remote working. Furthermore, research using electronic healthcare records has been conducted that suggests a drop in referrals across primary and secondary mental health services at both regional and national levels, with remote mediums being increasingly used for clinical contacts.

**Added value of this study**

This is one of the first studies looking at the impact of COVID-19 on access and service delivery in primary care psychological therapy services. Specifically, examining referrals and the sociodemographic and clinical characteristics of these as well as appointments and how appointments were delivered. We observed reductions in referrals during March 2020 lockdown, with some evidence potentially indicating changes in sociodemographic and clinical characteristics of referrals. This may suggest changes in demand amongst different groups. The impact of COVID-19 on the total number of appointments was limited, with a shift to remote care. This possibly suggests that the care of patients who sought treatment or were already in contact with services, may have only seen small disruptions.

**Implications of all the available evidence**

Although the observational nature of the data should be noted, the research has the potential to support planning of clinical practice and policy. Despite service providers in the present study appearing to adapt by offering remote care, there was a reduction in access to mental health services compared to what would have been expected at that time of year. This reduction has likely left some people without adequate mental health support, particularly as a switch to remote care may not have occurred rapidly across all of England. Although speculative, this deficit may result in greater pressures to treat a possible backlog as well as dealing with the potential aftermath of the long-term consequences of the pandemic on mental health.

Alt-text: Unlabelled box

## Introduction

In public healthcare systems such as the National Health Service (NHS) in England, primary care services are often the first port of call for patients with common mental health problems. Patients show a preference for psychological therapy over medication [1]. In England, psychological therapy in primary care settings is predominantly delivered by Improving Access to Psychological Therapy (IAPT) services [2]. IAPT services deliver a range of low- and high-intensity psychological interventions for depression and anxiety [2]. IAPT have implemented routine data collection, measuring sociodemographic and clinical patient characteristics as well as treatment data [2]. These data are nationally reported on a monthly basis [3]. In 2019–20 IAPT received approximately 1•69 million referrals [3].

There is growing concern about the profound and long-lasting impact of COVID-19 on mental health from multiple areas including academia, healthcare, and live experience advocates 0.^4^ Research suggests that clinically significant levels of mental distress rose during the pandemic in England – from approximately 19% in 2018–9 to 27% in 2020 [5]. People who have previously or are currently suffering from mental health conditions, as well as those who become mentally unwell during the pandemic, may potentially be vulnerable groups [4]. The pandemic may also disproportionately affect the mental health of other groups, including those with pre-existing mental and physical health conditions, individuals facing financial instability, ethnic minority groups, as well as young and older adults [4], [5], [6], [7], [8]. The provision of adequate mental health support to address the psychological impact of the pandemic and meet mental health needs is critical.

Despite the growing concerns about COVID-19 on mental health, less focus has been placed on how individuals with mental health problems are supported [9]. Concerns have been raised about adequate service provision during the pandemic, with staff shortages and service reconfigurations as well as the pressures of implementing infection control measures posing challenges to mental health staff [4,9]. Research suggests that referrals to primary and secondary mental health services reduced after lockdown regionally and nationally, with an increase in remote mediums to conduct clinical contacts [10], [11], [12]. However, less is known about how the impact on psychological care provision varied by patient characteristics.

The use of electronic healthcare records provides a first avenue to examine the impact of COVID-19 on primary care mental health services at scale [4]. Using electronic healthcare records from routinely collected data in IAPT services, we aim to understand service use during the pandemic. Specifically, we investigate access to psychological therapy services generally as well as how this may have varied by patient characteristics, early during the pandemic. Furthermore, we aim to understand the impact of COVID-19 on how clinical care was impacted and delivered.

## Methods

### Settings & design

IAPT are primary care services in England delivering psychological interventions for depression and anxiety [2]. A minimum dataset is routinely collected for all patients, recording data relating to patient characteristics, treatment, as well as routine measurement of clinical questionnaires for depression and anxiety [2]. The present study examines data from five NHS trusts in England, which were chosen for convenience purposes. All incoming referrals between the 1st January 2019 until 24th May 2020 were examined as well as all appointments from these referrals occurring between 1st January 2020 until 24th May 2020, covering the first national lockdown in England. The lockdown in England was implemented on the 23rd March [13]. All members of the public were asked to stay at home and not leave their house other than to shop for basic necessities, medical reasons, one form of exercise a day, or travelling to and from work in instances where this was absolutely necessary [13]. All shops selling non-essential goods were instructed to close and social events and gatherings were prohibited.^11^All data were extracted and fully anonymised by WW from Mayden, the software providers of the patient management system used by a large proportion of IAPT services. The authors CBS, KSB, and JF were provided with the dataset on 28th May 2020 and have access for ten years thereafter.

### Ethical approval

Due to the anonymous nature of the data, the present study was exempt from NHS Ethical Review. The project received ethical approval by the University of Bath (PREC: 19–015). Due to the anonymous nature of the data, it was not possible to retrieve individual patient consent. However, patients who had records indicating they did not want their data to be used for further processing were not included in the dataset provided by Mayden.

### Measures

To examine the impact of COVID-19 on access to services, we examined the data of all incoming referrals to IAPT. Specifically, we consider the total number of referrals as a measure of the impact of COVID-19 on patients accessing services. We further examined the characteristics of these referrals to examine changes in the demography or means of accessing services. Specifically, we examine characteristics including age, gender, ethnicity, the Index of Multiple Deprivation (IMD) as a proxy for socioeconomic status, population density (people per square kilometre) as a proxy for urbanicity, and referral source [14,15]. IMD and population density were determined at the Lower Super Output Area level via linkage to the Office of National Statistics data. Clinical characteristics of referrals were also explored, examining comorbid long-term health condition status, number of previous referrals, baseline depression measured via the Patient Health Questionnaire-9 (PHQ-9), and baseline anxiety measured by the Generalised Anxiety Disorder Scale-7 (GAD-7) [16,17]. In order to examine the impact of COVID-19 on service delivery, we examined appointment data, specifically the number of appointments and the consultation medium for attended appointments. Variable definitions can be found in Supplementary Material A.

### Statistical analysis

Descriptive time series are presented containing the weekly total count for categorical variables and weekly averages for continuous variables. Variables were examined individually, with the exception of ethnicity and referral source. Ethnicity was stratified by referral source as self-referrals were introduced into IAPT to increase access for minority groups such as Black, Asian and minority ethnic (BAME) groups [18]. To quantify changes in access, weekly counts of incoming referrals for the 9 weeks after lockdown were compared to the corresponding weeks in 2019.

Missing data for factor variables were defined as an additional factor level classed as *unknown*. Missing data for continuous variables were excluded. At the patient-level, the frequency of missing data for IMD and population density was approximately 0.5%. There may be various reasons as to why no baseline clinical measures are taken, including patients having never attended an appointment. For all reporting of the PHQ-9 and GAD-7, the last week of data (18th to the 24th May 2020) was excluded as it contained approximately 55% missing data, exceeding the maximum weekly missing data observed throughout the year. This possibly reflects that people referred close to the date of data extraction were unlikely to have had a first appointment booked within this short timeframe. After excluding the last week of data, the patient-level data contained approximately 28% missing data for the baseline PHQ-9 and GAD-7.

All analyses were performed in the R programming language [19]. All data analysis and visualisations were performed using base R and ‘ggplot2’. [19,20]

### Role of the funding source

The funders had no role in study design, data collection and analysis, decision to publish, or preparation of the manuscript.

## Results

### Sample characteristics

In the timeframe of 1st January 2019 to 24th May 2020, 171,823 referrals came into IAPT services across five different areas (Table 1). The majority of referrals were self-referrals (76%), typically female (66%), White (68%), with an average age of 38 years. However, patient characteristics demonstrate that services in different areas serve heterogeneous populations, with two serving populations from more urban areas, with slightly greater deprivation and a larger proportion of BAME individuals (Supplementary Material B). From the 1st January 2020 to 24th May 2020 248,628 appointments were scheduled amongst which the majority were attended (75%) and took place remotely (59%; Table 2).

**Table 1:** Characteristics of referrals from 1st January 2019 to 24th May 2020.

***N***	171,823
**Age**	37**•**94 (15**•**12)
**Gender -n (%)**	
Female	113,006 (65**•**8)
Male	58,643 (34**•**1)
Unknown	174 (0**•**1)
**Ethnicity -n (%)**	
White	116,964 (68**•**1)
Black, Asian and ethnic minority	32,600 (19**•**0)
*Asian*	16,976 (9**•**9)
*Black*	6857 (4**•**0)
*Mixed*	4913 (2**•**9)
*Other*	3854 (2**•**2)
Unknown	22,259 (13**•**0)
**Index of Multiple Deprivation**	21**•**02 (11**•**81)
**People per Square Kilometre**	7311**•**40 (7306**•**41)
**Long-Term Condition Status -n (%)**	
Long-Term Condition	49,562 (28**•**8)
No Long-Term Condition	97,985 (57**•**0)
Unknown	24,276 (14**•**1)
Number of previous referrals	1**•**01 (1**•**68)
Baseline PHQ-9*	14**•**39 (6**•**43)
Baseline GAD-7*	12**•**79 (5**•**42)
**Referral Source -n (%)**	
Self	130,089 (75**•**7)
Primary Care	32,672 (19**•**0)
Other	9062 (5**•**3)
Data is presented as mean (standard deviation) unless otherwise specified. PHQ-9: Patient Health Questionnaire −9; GAD-7: Generalised Anxiety Disorder Scale −7. *Data present until the 17th May 2020

**Table 2:** Characteristics of appointments from 1st January 2020 to 24th May 2020.

***N***	248,628
**Attendance**	
Attended	185,150 (74**•**5)
Cancelled by patient	21,333 (8**•**6)
Cancelled by provider	12,518 (5**•**0)
Did Not Attend or Late	29,627 (11**•**9)
**Consultation Medium of Attended Appointments**	
Face-to-Face	68,777 (37**•**1)
Remote	109,767 (59**•**3)
Other	4495 (2**•**4)
Unknown	2111 (1**•**1)
Data is presented as n (%).	

### Total referrals

**Fig. 1:**
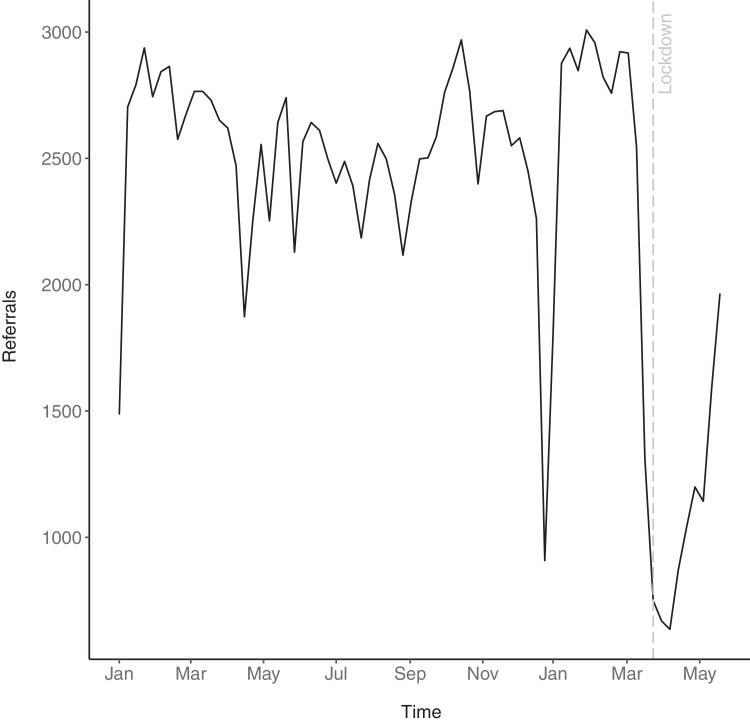
Total weekly referrals from 1st January 2019 to the 24th May 2020.

There was a decline in referrals in March 2020 (Fig. 1). The decline in referrals commenced approximately one week prior to the official government announcement of a lockdown in England, beginning on 23rd March 2020. The decline in referrals, relative to those observed at the same time in 2019, was greatest in the immediate three weeks of lockdown, reaching a maximum reduction of 74%. The decline in referrals is of a similar magnitude to a decline observed at the end of December during the Christmas holidays, albeit slightly larger.

Referrals started to gradually increase again over time. However, referrals had not fully recovered by the end of May. The total number of referrals in the week commencing on 18th May were still 28% lower than the corresponding week in 2019; that is, 72% of their usual volume.

In the early weeks after England entered lockdown, there was an average 55% reduction in referrals compared to the corresponding weeks in 2019 (Supplementary Material C). In the present dataset, this translated into approximately 12,000 fewer patients accessing mental health services than might be expected for that time of year.

### Sociodemographic characteristics of referrals

There are no clear changes in referrals by gender or long-term condition status (Supplementary Material D).

**Fig. 2:**
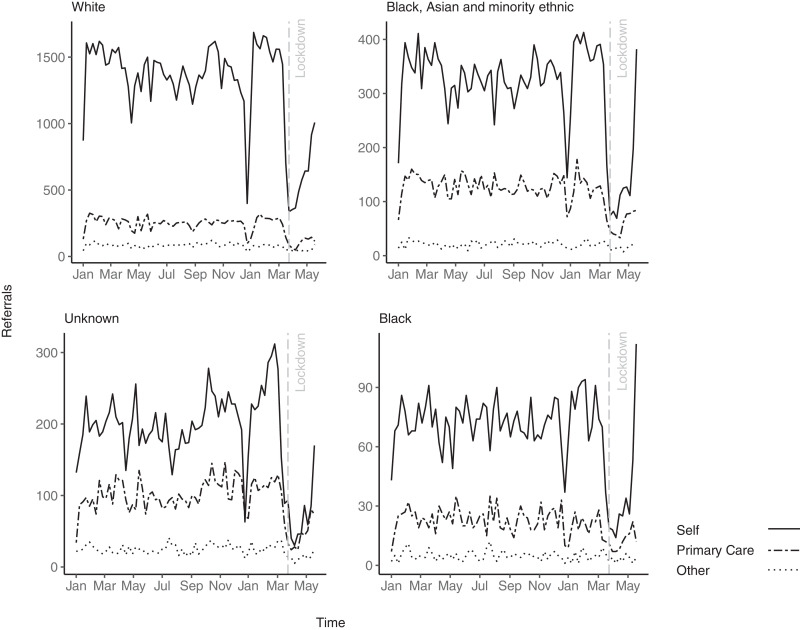
Total weekly referrals by ethnicity and referral source from 1st January 2019 to 24th May 2020.

There was a reduction in referrals across all referral sources and ethnicities after the lockdown was imposed in March 2020 (see Fig. 2). Self-referrals and referrals from other sources returned to baseline fastest, while referrals from primary care were increasing at a slower rate across all ethnicities. Compared to self-referrals from a White background, BAME self-referrals appeared to increase again at a faster rate after the initial drop observed around lockdown, being slightly higher than the corresponding time point in 2019. There were 382 self-referrals from patients with a BAME in the week commencing the 18th May 2020 compared to 338 in the corresponding week in 2019.When examining BAME subgroups, there was a particular increase in referrals towards the end of May by patients with a Black ethnic background, reaching the highest number of self-referrals observed across the entire timespan. There were 112 Black self-referrals in the week commencing the 18th May 2020 compared to 78 in the corresponding week in 2019.

**Fig. 3:**
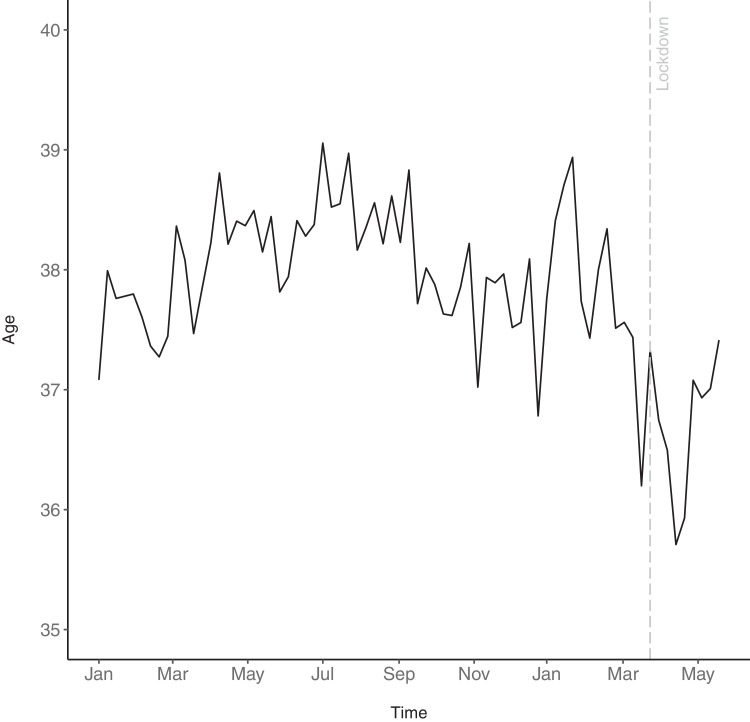
Weekly average age of referrals from 1st January 2019 to 24th May 2020.

It appears that age shows a time trend, where the average age increases during the summer and decreases again towards the winter (Fig. 3). There appeared to be a slight decrease in average age at referral in the 9 weeks after lockdown (36.8 years), when compared to the same time in 2019 (38.3 years). Although appearing to return to average levels, the average age had not returned to expected levels for the given time of year in May 2020.

**Fig. 4:**
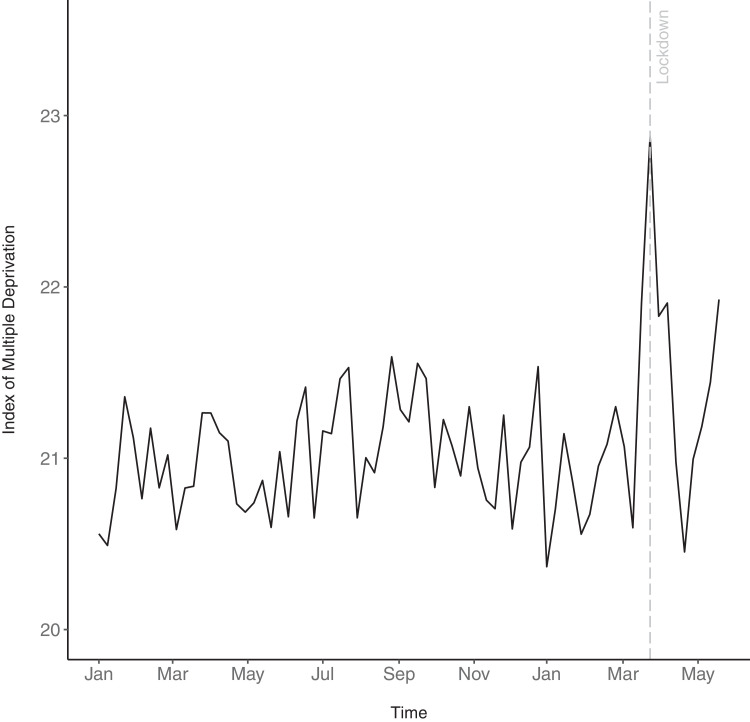
Weekly average Index of Multiple Deprivation of referrals from 1st January 2019 to 24th May 2020.

There appeared to be a brief spike in IMD early during lockdown (Fig. 4). This increase in IMD appeared to return to levels observed throughout the year relatively fast, with the average IMD of referrals in the 9 weeks post-lockdown being only marginally higher (21.5) compared to the same time in the previous year (21.0). However, it appears that IMD levels may be rising towards the end of May (Fig. 4).

**Fig. 5:**
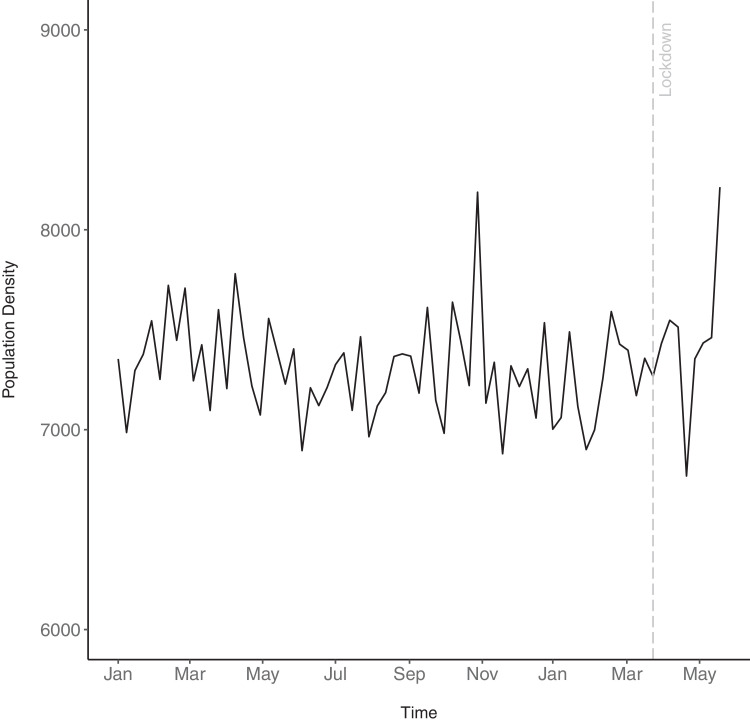
Weekly average population density of referrals from 1st January 2019 to 24th May 2020.

While there does not appear to be an obvious change in population density immediately after the lockdown was imposed (7515 people per Km[2] versus 7408 people per Km[2] for the same time in 2019), it appears that the population density of referrals may be increasing towards the end of May 2020 (Fig. 5).

### Clinical characteristics of referrals

**Fig. 6:**
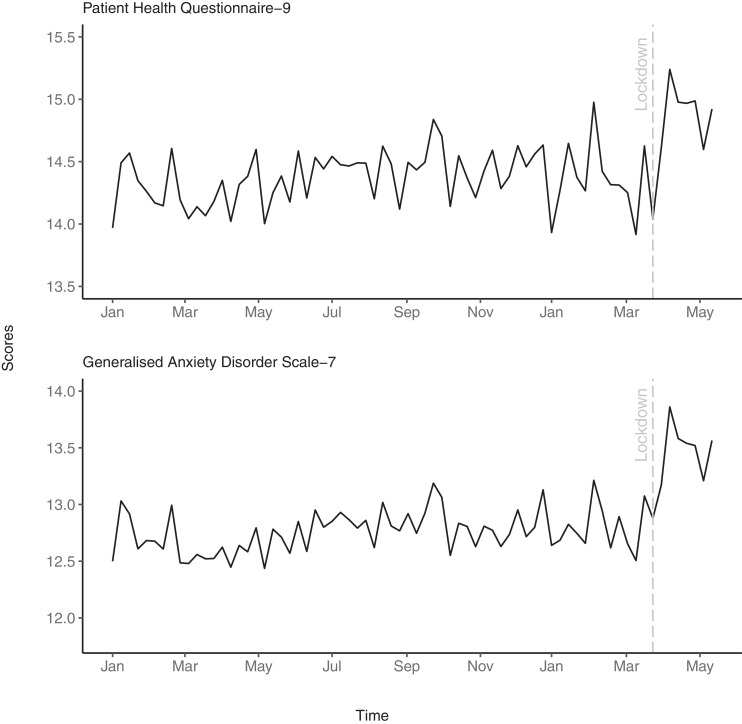
Weekly average baseline depression and anxiety scores for incoming referrals from 1st January 2019 to 17th May 2020.

There appears to be a slight increase in average PHQ-9 and GAD-7 immediately after lockdown was imposed (Fig. 6), with respective scores of 14.8 and 13.4 in the weeks following lockdown compared to 14.3 and 12.6 in the same time in 2019. This increase appeared to be sustained throughout lockdown, until May 2020

**Fig. 7:**
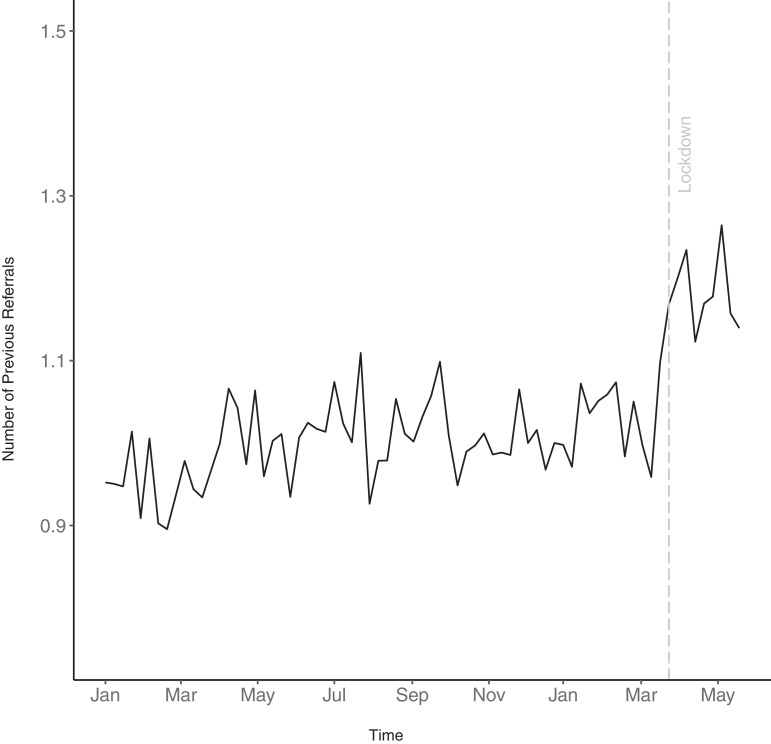
Weekly average number of previous referrals from 1st January 2019 to 24th May 2020.

There appeared to be a small increase in the average number of previous referrals in the 9 weeks after lockdown was imposed (Fig. 7),with patients having an average of 1.2 previous referrals post-lockdown compared to 1.0 over the same time the previous year .

### Appointments

**Fig. 8:**
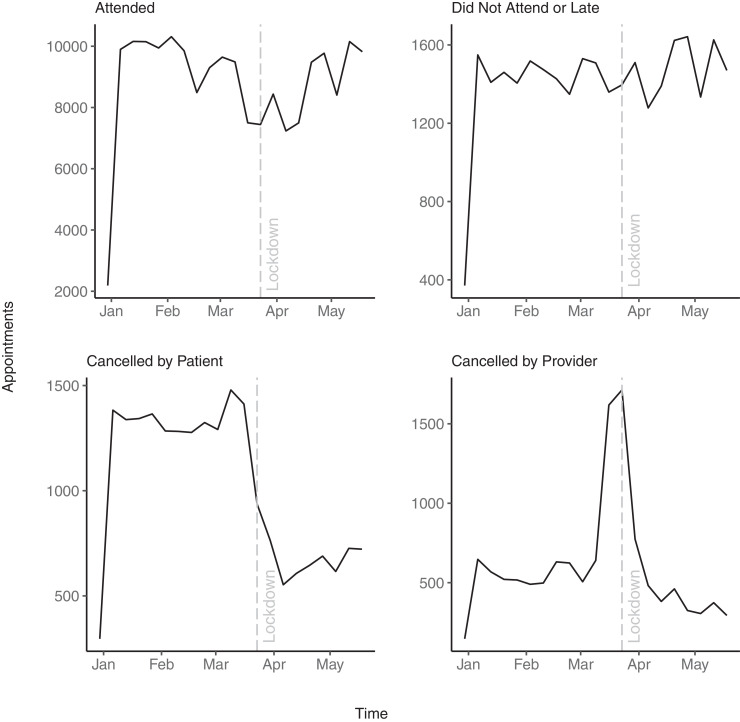
Weekly total appointments by attendance from 1st January 2020 to 24th May 2020.

There appears to be a brief, relatively small dip in attended appointments around lockdown. There appear to be no meaningful change in Did Not Attend (DNA) appointments or attended too late to be seen after lockdown. There was a relatively large reduction in appointments cancelled by patients after lockdown, whereas there was a brief spike in appointments cancelled by providers around lockdown (see Fig. 8).

Out of all attended appointments, face-to-face consultations reduced after the lockdown was imposed, with remote consultations increasing. Prior to lockdown, 64,201 (60•0%) of appointments took place *face-to-face* and 39,985 (37•4%) were recorded as *remote*. After lockdown, the majority of appointments took place *remotely* (69,782, 89•2%) with 4576 (5•8%) being recorded as *face-to-face* appointments. There was also an increase in appointments labelled *other* as well as a short spike in consultation mediums labels being *unknown*. Prior to lockdown, 1845 (1•7%) appointments had a consultation medium labelled as *other* and 882 (0•8%) were *unknown*. After lockdown, appointments with the label *other* rose to 2650 (3•4%) and 1229 (1•6%) had an unknown consultation medium.

## Discussion

Using electronic healthcare records, we examined the short-term impact of COVID-19 on primary care psychological therapy services in England, with regard to access and service delivery. There was a clear drop in referrals to IAPT around the implementation of lockdown, resulting in approximately 55% fewer patients accessing services in the early weeks after the lockdown was imposed in the UK. There appeared to be a trend indicating faster increases in the number of referrals from BAME, especially Black patients, once referrals started to increase again. While the changes were relatively small, there was some evidence to suggest an increase in referrals from younger patients, patients living in higher deprivation and urban areas, those with higher baseline depression and anxiety scores as well as those who have previously sought treatment. Despite reductions in the number of people accessing services, it appears that the care of patients receiving treatment showed somewhat short-lived disruptions, with services quickly moving to provide remote consultations.

Overall, there was an average reduction of 55% in referrals in the early weeks after lockdown when compared to the same timeframe in 2019. This decline in referrals began approximately one week prior to lockdown and reached the maximum level within three weeks after the lockdown was announced, with a 74% reduction of referrals in 2020 compared to the same time in 2019. This appears somewhat consistent with regional reports as well as the national trend observed through the monitoring of activity in the patient management software used by a majority of IAPT services, where referrals dropped by approximately 70% early after lockdown was announced [[10], [11], [12],^21^]. The use of primary care psychological therapies are similar to those observed in other health services, such as a reduced number of patients accessing Accident and Emergency Departments and General Practice [22,23]. Although not returning to baseline, the number of referrals appeared to gradually increase again over time, with referrals being at 72% of their usual volumes. Albeit slightly lower, a similar pattern is observed at a national level, where referrals in July 2020 were at 60% of the volumes observed prior to COVID-19 [21]. Over time, the referrals appeared to increase again to a greater degree with national data showing that referrals to adult mental health services, including IAPT, were approximately 10% lower when examined over a greater period from April 2020 to August 2020 compared to the same time in 2019.

Despite referral rates increasing again as the lockdown progressed, a deficit in referred patients was observed. If the present research is extrapolated across England, with an assumed 1.69 million referrals per year, as observed in 2019–20, approximately 160,900 patients who may have normally been referred did not access mental health services in the first weeks after lockdown was imposed [3]. However, this deficit estimate is likely conservative – figures may be higher in the longer term as referrals had not returned to baseline towards the end of May and the proposed figure does not account for a possible increase in mental health needs or the annual increase in referrals – from 2018/19 to 2019/2020 the annual increase in referrals was 5.7%[3,5]

Self-referrals appeared to have recovered from the effects of lockdown most rapidly, which may have been facilitated through an increase in the availability and use of online referral systems [21]. Online self-referrals to IAPT were 13% higher in July 2020 than pre-lockdown [21]. This recovery of self-referral rates was most pronounced in BAME groups, which appeared to return to baseline most rapidly after the initial observed decline. It is tentatively suggested that this could reflect a greater impact of COVID-19 on the mental health of the BAME community, which would be consistent with emerging findings highlighting inequalities of COVID-19 and a higher percentage of people from a BAME background reporting worse than usual mental health – approximately 50% compared to 35% across all adults [24,25]. However, it also indicates a greater opportunity for BAME groups to access mental health services and may speak to the efficacy of opening up services through self-referrals as a means of doing so [18]. Population-based surveys suggest a higher number of young adults reporting worse mental health compared to prior to COVID-19 as well as higher levels of depression and anxiety during lockdown amongst people from low-income households, which may be related to unstable housing and jobs or life transitions, amongst others, that may have been exacerbated as a result of lockdown [25]. Similarly, in the present study, we observed an increase in average IMD and a decrease in age at referral, which may indicate an increased demand for psychological therapy by these groups. Population-based surveys have further identified higher levels of depression and anxiety during lockdown amongst people living in urban areas [24]. We found some evidence to suggest that the population density of referrals was increasing over time. This could potentially be a result of a higher impact of COVID-19 in these areas, with more urban areas showing a higher age-standardised mortality rate of COVID-19 between 1st March to 31st July 2020 compared to more rural settings [26].

There was an slight change in the clinical severity of referrals, with an increase in average depression and anxiety scores of incoming referrals after the lockdown had been implemented in the UK. This increase remained somewhat stable throughout lockdown. Due to the observational nature of the data, it is difficult to discern whether increases in depression and anxiety resulted from a rise in symptoms amongst the general population or whether patients with more severe symptoms were accessing services to a greater degree after the lockdown was imposed. However, population-based surveys showed a decrease in both depression and anxiety from the start of lockdown until May [24]. As such, the latter is suggested as more probable. Furthermore, there was a small increase in the number of previous referrals after lockdown, suggesting that patients who had already accessed services previously were returning to a greater degree. There is some evidence to suggest that patients who re-refer present with more complex psychological problems and may be more likely to have higher baseline depression and anxiety [27].

While there was an evident impact of COVID-19 lockdown on people accessing primary mental services, it appears that service delivery for patients already being treated pre-lockdown or starting treatment during lockdown may have only seen limited disruption as services appeared to adapt quickly to new practices. This is consistent with national trends showing no dramatic drop in clinician activity recorded by the IAPT patient management software [21]. Services appeared to rapidly adapt, implementing infection control measures by switching to remote consultations almost exclusively. Mental health staff accounts mirror this, reporting rapid innovation with a particular emphasis on remote working [9]. A small proportion of appointments were still recorded as face-to-face after lockdown. There may have been a clinical necessity to continue to see patients face-to-face. However, it is also likely a result of data error – service providers may not have had appropriate labels early during lockdown as patient management systems were being updated to reflect rapid transitions to new ways of working. An example of this might be the use of remote meeting software. This is consistent with an increased practice of labelling appointments with consultations medium classed as *other* and *unknown*. However, it should be noted that there is a sparsity of patients reports regarding their experiences of accessing and using mental health services during the pandemic.

To our knowledge, the present study is one of the first to examine a quantifiable impact of COVID-19 on primary care mental health services at scale, with data from a wide and diverse range of service providers across multiple geographic regions in England. Nonetheless, the dataset contains only a small subset of service providers in England and may therefore not be nationally representative. There may be variation by service providers and regions that is not captured by the data used in the present study. However, the observed trends appear consistent with those detected by the monitoring of activity within IAPT service's patient management software [21]. The nationally reported IAPT data will provide further insights in the long-term and has the benefit of a larger sample that is representative of all service providers; however, the present analysis allows for more detailed insights. It should be noted that the present research is using observational data, taking a descriptive approach. As such, it is not possible to draw causal conclusions, and the capacity of estimating future impact is limited and remains speculative. We also made no adjustments to accommodate the annual increase in referrals. While this has the benefit of taking into account seasonal variations, which appear to exceed annual increases in magnitude, it potentially leads to deficit figures being underestimated. The figure may further be underestimated as mental health needs may have increased during the pandemic [5]. We also only examine appointment data from referrals occurring in 2019/2020. Furthermore, we cannot quantify the influences of broader organisational and societal events that may have influenced mental healthcare. For example, the NHS ran an ‘Open for Business’ campaign promoting the public to access healthcare, which may have increased confidence in people seeking treatment [28]. Similarly, organisational influences could include staffing levels and/or service restructuring that influence the capacity to deliver healthcare. Despite occurring after the timeframe of the present study, and thus having little influence on the present findings, protests in support of the ‘Black Lives Matter’ movement occurred during Spring/Summer of 2020 concomitant to the pandemic. This will be an important factor to consider in future research as it may disproportionately affect minority groups.

A reduction of referrals took place during the early stages of COVID-19, producing approximately a 55% deficit in patients receiving mental healthcare. A concern may be that a backlog of patients has accumulated, which may cause future pressures on service providers to treat these patients in addition to a possible excess of patients who may seek mental health support as the long-term consequences of COVID-19 become more apparent. Given the faster increase in self-referrals after the initial drop from BAME groups compared to others, as well as a potential trend showing increases in deprivation and urbanity of referrals, service providers catering to these populations may experience a particular surge in demand. Services may also see changes in the demography of referrals such as a higher proportion of younger patients, patients who have previously sought treatment, or patients with a higher clinical severity. This may serve as a reminder of the need for cultural competency in psychological therapy to meet the needs of all patients accessing services [29]. Periodical horizon scanning of the demography of patients accessing services may provide an avenue to assure that developments in cultural competency adequately reflect demographic changes.

Despite access to mental health services being impacted by COVID-19, the data suggests that service providers in the present study were able to adapt to the pandemic with the adoption of remote consultations. This shift likely provided essential continuity of care to patients in receipt of mental healthcare. Previous research suggests that remote Cognitive Behavioural Therapy is effective and may increase treatment adherence [30], [31], [32]. However, approximately 40% of community and psychological therapy staff have reported difficulties with learning new technologies too quickly or without enough training and experiencing technical difficulties with remote consultation [9]. Furthermore, remote therapy may come at the cost of poorer maintenance after treatment [32]. Early evidence from the nationally reported data shows a brief dip in clinical outcomes between March to April 2020 but an increase in clinical outcomes in June – July 2020; reaching higher levels than prior to COVID-19 [33]. The long-term effects on clinical outcomes remain to be determined, with a pressing need for future research.

The present findings provide insight into the short-term impact of the COVID-19 pandemic on psychological therapy services. Due to the observational nature of the data, results should be interpreted with caution. However, they have the potential to support the planning of clinical practice and public health policy; particularly as restriction are likely to remain in place until national vaccination programmes have been completed but also managing the aftermaths of a pandemic. The current findings also highlight a need for further research, presenting avenues for future directions. The long-term impact of COVID-19 on mental health services and mental health more generally remains to be determined as the delayed consequences, such as economic hardship, become more apparent.

## Authors’ contributions

Clarissa Bauer-Staeb – Conceptualisation, Methodology, Writing – Original Draft, Formal Analysis, Visualisation.

Alice Davis - Conceptualisation, Methodology, Writing – Review & Editing.

Theresa Smith - Conceptualisation, Writing – Review & Editing.

Emma Griffith - Writing – Review & Editing, Supervision.

Chris Eldridge - Resources, Writing – Review & Editing.

Wendy Wilsher - Data Curation.

David Betts – Data Curation, Resources, Writing – Review & Editing.

Julian Faraway - Conceptualisation, Methodology, Formal Analysis, Writing – Review & Editing, Supervision.

Katherine Button - Conceptualisation, Methodology, Writing – Review & Editing, Supervision.

## Funding

AD and TS were funded by Innovate UK (KTP #11105). The funders had no role in study design, data collection and analysis, decision to publish, or preparation of the manuscript.

## Data sharing statement

While the data are anonymous, they are nonetheless subject to restrictions. The authors were given permission to process the data, but the data ownership remains with the NHS. As such, the authors cannot make any data publicly available. Any permission to access NHS data must be made through dedicated channels.

## Declaration of competing interest

CBS, JF, KB, TS, AD, DB, WW, CE report no conflicts of interest. EG reports grants from the British Psychological Society outside the submitted work and is a member of the British Psychological Society, Division of Clinical Psychology, Digital Healthcare.

## Appendix Supplementary materials

Image, application 1
