# Supplementary material for: The early impact of COVID-19 on primary care psychological therapy services: A descriptive time series of electronic healthcare records
Source: eClinicalMedicine. 2021 Jun 4;37:100939. doi: 10.1016/j.eclinm.2021.100939 (PMC8343255; doi:10.1016/j.eclinm.2021.100939)

**SUPPLEMENTARY MATERIALS**

**Supplementary Material A – Variable definition**

Appointment attendance, consultation medium and ethnicity were grouped based on theoretical grounds. Appointment attendance was classified on the basis of patients having clinical contact, with cancellation groupings remaining the same. Consultation medium was classified on the basis of appointments taking place in person or remotely, with a separate level when it was not possible to determine this distinction. Ethnicity was divided into majority and minority ethnic groups. Referral source was reduced on the basis on available data points for each level, retaining the original labels for the two primary referral sources. Missing data was labelled as unknown if there was no entry recorded or the data entry indicated that the measurement was not available.

**Supplementary Table A1. Variable definition**

| **Variable** | **Class** | **Levels** | **Data Source** | **Measurement** |
| --- | --- | --- | --- | --- |
| **Referral-Level Data** |  |  |  |  |
| Referrals | Count | N.A. | IAPT MDS | System capture |
| Referral Source | Categorical | Self-Referral | IAPT MDS | Clinician report |
|  |  | Primary Care |  |  |
|  |  | Other |  |  |
|  |  | *Local Authority Services* |  |  |
|  |  | *Employer* |  |  |
|  |  | *Justice System* |  |  |
|  |  | *Child Health* |  |  |
|  |  | *Independent/Voluntary Sector* |  |  |
|  |  | *Acute Secondary Care* |  |  |
|  |  | *Other Mental Health NHS Trust* |  |  |
|  |  | *Internal referrals from Community Mental Health Team (within own NHS Trust)* |  |  |
|  |  | *Internal referrals from Inpatient Service (within own NHS Trust)* |  |  |
|  |  | *Transfer by graduation (within own NHS Trust)* |  |  |
|  |  | *Other* |  |  |
|  |  | *IAPT* |  |  |
| Age | Continuous | N.A. | IAPT MDS | Self-report |
| Gender | Categorical | Female | IAPT MDS | Self-report |
|  |  | Male |  |  |
|  |  | Unknown |  |  |
|  |  | *Not known* |  |  |
|  |  | *Not specified* |  |  |
|  |  | *Missing* |  |  |
| Ethnicity | Categorical | White | IAPT MDS | Self-report |
|  |  | Black, Asian and ethnic minority |  |  |
|  |  | *Black* |  |  |
|  |  | *Asian* |  |  |
|  |  | *Mixed* |  |  |
|  |  | *Other* |  |  |
|  |  | Unknown |  |  |
|  |  | *Not Stated* |  |  |
|  |  | *Not Known* |  |  |
|  |  | *Missing* |  |  |
| Long-Term Condition Status | Categorical | Long-Term Condition | IAPT MDS | Self-report |
|  |  | No Long-Term Condition |  |  |
|  |  | Unknown |  |  |
|  |  | *Unknown (Person asked and does not know or is not sure)* |  |  |
|  |  | *Not Stated (Person asked but declined to provide a response)* |  |  |
|  |  | *Missing* |  |  |
| Previous Referrals | Continuous | N.A. | IAPT MDS | System capture |
| Baseline Patient Health Questionnaire-9 (PHQ-9) | Continuous | N.A. | IAPT MDS | Self-report |
| Baseline Generalised Anxiety Disorder Scale-7 (GAD-7) | Continuous | N.A. | IAPT MDS | Self-report |
| Index of Multiple Deprivation (IMD) | Continuous | N.A. | ONS database | Self-report |
| People per Km^2^ | Continuous | N.A. | ONS database | Self-report |
| **Appointment-Level Data** |  |  |  |  |
| Appointments | Count | N.A. | IAPT MDS | System capture |
| Attendance |  | Attended | IAPT MDS | Clinician report |
|  |  | *Attended on time or, if late, before the relevant care professional was ready to see the patient* |  |  |
|  |  | *Arrived late, after the care professional was ready to see the patient, but was seen* |  |  |
|  |  | Did not attend or Late |  |  |
|  |  | *Patient arrived late and could not be seen* |  |  |
|  |  | *Did not attend - no advance warning given* |  |  |
|  |  | Cancelled by patient |  |  |
|  |  | Cancelled by provider |  |  |
| Consultation Medium | Categorical | Face-to-face | IAPT MDS | Clinician report |
|  |  | Remote |  |  |
|  |  | *Telephone* |  |  |
|  |  | *Telemedicine web camera* |  |  |
|  |  | *Email* |  |  |
|  |  | *Short Message Service (SMS)* |  |  |
|  |  | Other |  |  |
|  |  | *Other* |  |  |
|  |  | *Talk type for a Person unable to speak* |  |  |
|  |  | Unknown |  |  |
|  |  | *Missing* |  |  |
| N.A. – Not Applicable; IAPT MDS - Improving Access to Psychological Therapies Minimum Dataset; ONS - Office of National Statistics | | | | |

**Supplementary Material B – Descriptive characteristics stratified by NHS trust**

| ***Supplementary Table B*. Characteristics of referrals from 1st January 2019 to 24^th^ May 2020 stratified by NHS Trust** | | | | | |
| --- | --- | --- | --- | --- | --- |
|  | ***Trust 1*** | ***Trust 2*** | ***Trust 3*** | ***Trust 4*** | ***Trust 5*** |
| ***n*** | 25,980 | 28,828 | 37,593 | 47,331 | 32,091 |
| **Age** | 38**·**13 (15**·**17) | 41**·**09 (16**·**19) | 35**·**95 (13**·**64) | 37**·**47 (15**·**42) | 38**·**01 (14**·**85) |
| **Gender -n (%)** | |  |  |  |  |
| Female | 16,930 (65**·**2) | 18,486 (64**·**1) | 24,909 (66**·**3) | 31,144 (65**·**8) | 21,537 (67**·**1) |
| Male | 9,022 (34**·**7) | 10,331 (35**·**8) | 12,636 (33**·**6) | 16,108 (34**·**0) | 10,546 (32**·**9) |
| Unknown | 28 (0**·**1) | 11 (0**·**0) | 48 (0**·**1) | 79 (0**·**2) | 8 (0**·**0) |
| **Ethnicity -n (%)** | |  |  |  |  |
| White | 22,386 (86**·**2) | 24,908 (86**·**4) | 16,195 (43**·**1) | 3,9987 (84**·**5) | 13,488 (42**·**0) |
| Black, Asian and ethnic minority | 1,049 (4**·**0) | 1,329 (4**·**6) | 14,576 (38**·**8) | 2,491 (5**·**3) | 13,155 (41**·**0) |
| Unknown | 2,545 (9**·**8) | 2,591 (9**·**0) | 6,822 (18**·**1) | 4,853 (10**·**3) | 5,448 (17**·**0) |
| **Index of Multiple Deprivation** | 17**·**70 (12**·**15) | 15**·**79 (11**·**08) | 25**·**28 (10**·**70) | 21**·**41 (12**·**71) | 22**·**82 (9**·**27) |
| **People per Square Kilometre** | 2,878**·**67 (2653**·**96) | 3,071**·**35 (2680**·**27) | 14,676**·**56 (9303**·**41) | 4,638**·**41 (3755**·**63) | 10,005**·**72 (6163**·**44) |
| **Long-Term Condition Status -n (%)** | | |  |  |  |
| Long-Term Condition | 6,575 (25**·**3) | 10,871 (37**·**7) | 8,902 (23**·**7) | 15,915 (33**·**6) | 7,299 (22**·**7) |
| No Long-Term Condition | 14,317 (55**·**1) | 16,716 (58**·**0) | 21,404 (56**·**9) | 28,244 (59**·**7) | 17,304 (53**·**9) |
| Unknown | 5,088 (19**·**6) | 1,241 (4**·**3) | 7,287 (19**·**4) | 3,172 (6**·**7) | 7,488 (23**·**3) |
| Referral Number | 2**·**41 (2**·**16) | 1**·**96 (1**·**50) | 1**·**85 (1**·**57) | 2**·**08 (1**·**58) | 1**·**81 (1**·**56) |
| Baseline PHQ-9* | 15**·**64 (6**·**29) | 13**·**65 (6**·**60) | 13**·**88 (6·56) | 15**·**07 (6**·**03) | 13**·**76 (6**·**48) |
| Baseline GAD-7* | 13**·**85 (5**·**08) | 12**·**12 (5**·**58) | 12·50 (5**·**67) | 13**·**09 (5**·**07) | 12**·**51 (5**·**54) |
| **Referral Source -n (%)** | |  |  |  |  |
| Self | 17,502 (67**·**4) | 25,935 (90**·**0) | 27,498 (73**·**1) | 40,329 (85**·**2) | 18,825 (58**·**7) |
| Primary Care | 7,330 (28**·**2) | 2,043 (7**·**1) | 8,778 (23**·**4) | 2,621 (5**·**5) | 11,900 (37**·**1) |
| Other | 1,148 (4**·**4) | 850 (2**·**9) | 1,317 (3**·**5) | 4,381 (9**·**3) | 1,366 (4**·**3) |
|  | | | | | |
| Data is presented as mean (standard deviation) unless otherwise specified. PHQ-9: Patient Health Questionnaire -9; GAD-7: Generalised Anxiety Disorder Scale -7. *Data until 17th May 2020. | | | | | |

**Supplementary Material C – Comparison of referrals in 2019 and 2020 before and after lockdown**

**Supplementary Table C. Total weekly referrals 9 weeks after lockdown compared between 2019 and 2020**

|  | **Referrals 2019** | **Referral 2020** | **Difference (n)** | **Difference (%)** |
| --- | --- | --- | --- | --- |
|  | 2,651 | 749 | -1,902 | -72 |
|  | 2,620 | 669 | -1,951 | -74 |
|  | 2,471 | 636 | -1,835 | -74 |
|  | 1,873 | 870 | -1,003 | -54 |
|  | 2,253 | 1,040 | -1,213 | -54 |
|  | 2,555 | 1,199 | -1,356 | -53 |
|  | 2,253 | 1,143 | -1,110 | -49 |
|  | 2,642 | 1,588 | -1,054 | -40 |
|  | 2,740 | 1,965 | -775 | -28 |
|  |  |  |  |  |
| Sum/average |  |  | -12,199 | -55 |

**Supplementary Material D – Referrals by Sociodemographic Characteristics**

**Supplementary Figure D1. Average weekly referrals by gender from 1st January 2019 to 24^th^ May**

**
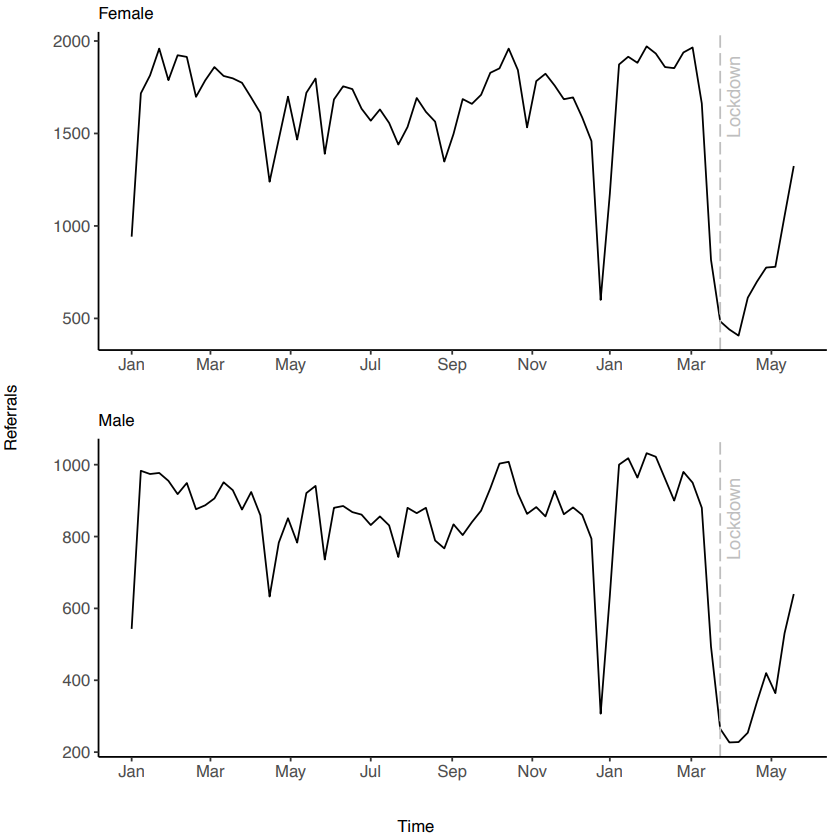
**

*Missing data for gender is omitted as its trivial

**Supplementary Figure D2. Total weekly referrals by long-term condition status from 1st January 2019 to 24^th^ May**


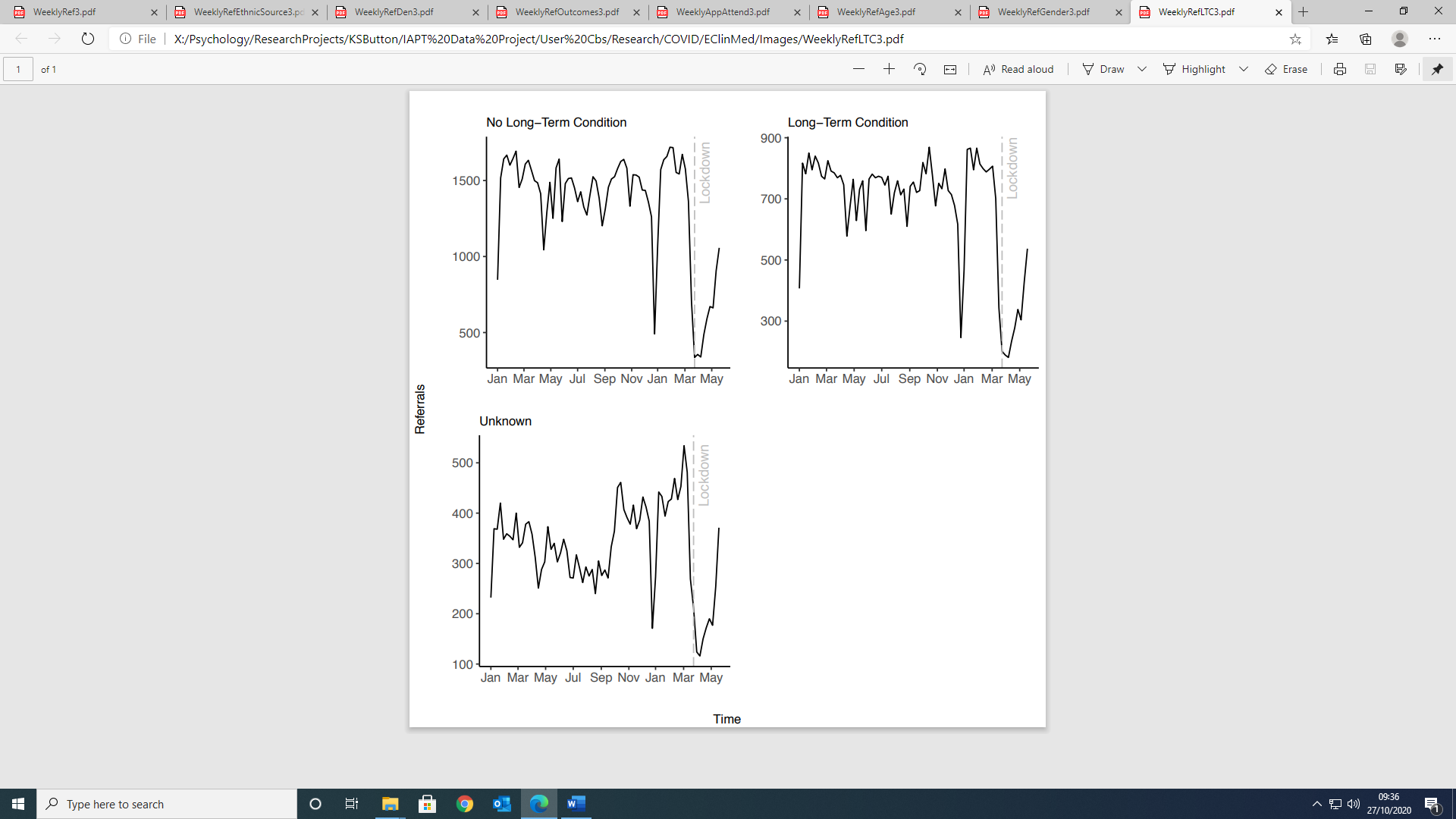

Supplement: Supplementary file 1 [file mmc1.docx]
